# A Descriptive Analysis of Mediterranean Diet Meal Plans Using the Dietary Inflammatory Index, Dietary Antioxidant Index, and Dietary Lipid Indices: Implications for Dietary Intervention for Metabolic Dysfunction-Associated Steatotic Liver Disease (MASLD) Research

**DOI:** 10.3390/nu18081281

**Published:** 2026-04-17

**Authors:** Melvin Bernardino, Claudio Tiribelli, Natalia Rosso

**Affiliations:** 1 Metabolic Liver Disease Unit, Fondazione Italiana Fegato, 34149 Trieste, Italy; ctliver@fegato.it; 2 Department of Life Science, University of Trieste, 34100 Trieste, Italy; 3 Department of Science and Technology, Philippine Council for Health Research and Development, Taguig 1631, Philippines

**Keywords:** metabolic dysfunction-associated steatotic liver disease, Mediterranean diet, dietary inflammatory index, dietary antioxidant index, dietary lipid index

## Abstract

**Background/Objectives**: Metabolic dysfunction-associated steatotic liver disease (MASLD) is a common chronic liver disorder linked to obesity, insulin resistance, and dyslipidemia. Nutrition plays a central role in modulating hepatic lipid metabolism, oxidative stress, and inflammation, yet practical, evidence-based dietary strategies remain limited. This study aimed to develop Mediterranean diet-based meal plans with varying macronutrient compositions and to characterize their nutritional profiles, as well as to evaluate them using established nutritional indices and diet score calculations, such as the Dietary Inflammatory Index, Dietary Antioxidant Index, and dietary lipid indices. **Methods**: Clinical practice guidelines (CPGs) from various academic and professional organizations were reviewed to assess current non-pharmacological treatments for MASLD, with a focus on determining whether the Mediterranean diet is the most recommended dietary pattern. Traditional, low-carbohydrate, and low-fat MedDiet patterns were translated into food-based meal plans. A 7-day meal plan was developed and analyzed for nutrient composition, then evaluated using the Dietary Inflammatory Index (DII), Dietary Antioxidant Index (DAI), Dietary Lipophilic Index (DLI), and Dietary Lipophilic Load (DLL). A Western diet (WD) that is characterized by ultra-processed food (UPF) was included as a comparative reference. **Results**: The validated dietary score calculations showed that all MedDiet patterns demonstrated consistently low DII scores (−2.00 to −2.81) and high DAI scores (3 to 20.03), whereas the WD showed high DII scores (5.0 to 6.09) and low DAI scores (−12.47 to −17.99). Despite these variations in macronutrients, the menu developed in the study on three MedDiet patterns showed negative DII and positive DAI scores. When comparing the traditional MedDiet with the WD, which have similar macronutrient distributions, the WD was characterized by less favorable DII and DAI scores. **Conclusions**: This study provides a descriptive, guideline-informed framework for Mediterranean diet-based meal plans with varying macronutrient compositions. Utilizing DII, DAI, DLI, and DLL offers a potential framework for designing dietary interventions. Further validation through clinical studies is needed to justify the potential for practical and digital translation. Nevertheless, the study provides initial insights that may inform future research on nutritional approaches for MASLD integrating dietary indices.

## 1. Introduction

Metabolic dysfunction-associated steatotic liver disease (MASLD) is a chronic liver disorder characterized by excessive lipid accumulation in hepatocytes in the absence of significant alcohol consumption [1]. Currently, MASLD affects approximately 38% of the adult population and 7% to 14% of the pediatric population [2]. The term MASLD has recently replaced non-alcoholic fatty liver disease (NAFLD) to more accurately reflect the central role of metabolic dysfunction, including obesity, insulin resistance, type 2 diabetes mellitus (T2DM), and dyslipidemia, in disease pathogenesis [3,4,5]. At the molecular level, MASLD is driven by profound alterations in hepatic lipid metabolism, characterized by increased free fatty acid influx, enhanced de novo lipogenesis, impaired beta-oxidation, and dysfunctional lipid export [6,7]. These disturbances are closely linked to mitochondrial dysfunction, resulting in reduced oxidative capacity and excessive production of reactive oxygen species (ROS) [8,9]. The accumulation of oxidative stress promotes lipid peroxidation, mitochondrial DNA damage, and activation of stress-responsive signaling pathways, thereby exacerbating hepatocellular injury [10,11]. Concurrently, MASLD is associated with chronic low-grade inflammation mediated by dysregulated cytokine production, activation of inflammatory transcription factors, and crosstalk between hepatocytes, immune cells, and adipose tissue [7,12]. Insulin resistance further amplifies these processes by promoting hepatic lipid accumulation and impairing metabolic flexibility [13]. Collectively, these interconnected molecular events drive the progressive transition from simple steatosis to inflammatory and fibrotic liver disease, underscoring MASLD as a complex, multi-system disorder with both metabolic and molecular underpinnings. Given the deep understanding of the disease pathophysiology, proper nutrition and diet along with exercise remains the most safe and effective strategy to prevent and manage MASLD [14,15,16].

Proper nutrition and diet plays a central role in the development and progression of MASLD by acting as a key upstream regulator of hepatic molecular pathways. Beyond its contribution to energy excess, dietary composition directly influences transcriptional and signaling networks that govern lipid metabolism, inflammation, and redox homeostasis within the liver [17]. As such, proper nutrition and dietary strategies represent a powerful non-pharmacological intervention in MASLD, providing a unifying framework for understanding disease pathogenesis and informing targeted nutritional strategies for prevention and treatment. An extensive body of scientific evidence has accumulated demonstrating the influence of dietary patterns on the prevention and management of MASLD. Although individual dietary approaches confer distinct metabolic benefits, each is also associated with specific limitations that warrant consideration in clinical practice. In the current literature, dietary patterns such as the Mediterranean diet (MedDiet) [18], low-carbohydrate diet (LCD) [19], ketogenic diet (KD) [20], dietary approach to stop hypertension (DASH) [21], low-fat diet (LFD) [22], intermittent fasting (IF) [23], and vegetarian diet (VD) [24] have been evaluated for their potential benefits in managing MASLD.

These dietary patterns have been evaluated for their beneficial effects on a broad range of metabolic and hepatic outcomes, including improvements in liver function tests, modulation of gut microbiota composition, attenuation of inflammatory and oxidative stress markers, and favorable regulation of glucose and lipid metabolism. Additional benefits have been reported for cardiometabolic parameters such as blood pressure and anthropometric measures, including reductions in body weight, body mass index (BMI), and waist circumference (WC). Among these approaches, the MedDiet remains the most extensively investigated and consistently recommended dietary pattern for the management of MASLD [17,25]. However, it is important to acknowledge that the MedDiet itself encompasses considerable heterogeneity, particularly with respect to macronutrient distribution. Variants such as the traditional MedDiet, low-carbohydrate MedDiet, and low-fat MedDiet differ in their relative proportions of carbohydrates and fats which may influence metabolic responses and clinical outcomes.

Aside from the type of dietary pattern and macronutrient composition, there is increasing attention on diet quality, specifically on how dietary patterns target the pathophysiological mechanism of the disease. Evidence from nutritional science indicates that specific dietary components play a role in regulating metabolic processes [26]. Indices such as the Dietary Inflammatory Index (DII), Dietary Antioxidant Index (DAI), and dietary lipid indices, including the Dietary Lipophilic Index (DLI) and Dietary Lipophilic Load (DLL), are validated nutrition tools that enable the evaluation of dietary quality by capturing the functional impact of diet on inflammatory, oxidative, and lipid-related processes. DIIs evaluate how diet affects inflammation, ranging from blood concentrations of inflammatory cytokines to chronic diseases [27,28]. DAIs evaluate the antioxidant capacity of diets based on the intake of major dietary antioxidants [29,30]. The DLI is a lipid quality measure that reflects how lipophilic the overall diet is on average. Meanwhile, the DLL is a lipid quantity measure that reflects the total amount of lipophilicity you take in from all fat sources [31].

In the context of MASLD, these nutritional indices have been increasingly investigated. Evidence suggests that lower DII scores may confer clinical benefits in pediatric MASLD, as anti-inflammatory diets have been associated with reduced severity of hepatic steatosis and fibrosis [26]. Furthermore, systematic reviews indicate that higher DII scores correlate with an increased risk of MASLD, while lower DII scores are linked to a decreased risk of disease development [32]. On the other hand, higher DAI scores were associated with better liver function, lipid profiles, and lower risk of hepatic fibrosis [33]. Lastly, studies on DLL and DLI in MASLD suggest that dietary fatty acid quality affects MASLD risk differently based on sex and body weight status. The DLI can serve as a predictive indicator for MASLD based on dietary fatty acid properties [31]. Together, these indices provide mechanistic insight into how diet modulates inflammation, oxidative stress, and lipid metabolism, core molecular processes underpinning MASLD.

Building on this framework, this study aims to develop a nutritionally optimized food-based diet plan that targets key pathophysiological mechanisms involved in MASLD. Specifically, the objectives are: (a) to review current clinical practice guideline recommendations for MASLD and translate these recommendations into a practical, food-based diet plan; (b) to design dietary patterns with varying macronutrient compositions to reflect different therapeutic nutritional strategies/options; and (c) to evaluate these dietary patterns using established nutritional indices/diet score calculations related to anti-inflammatory potential, antioxidant capacity, and lipid quality and quantity.

## 2. Materials and Methods

**Figure 1:**
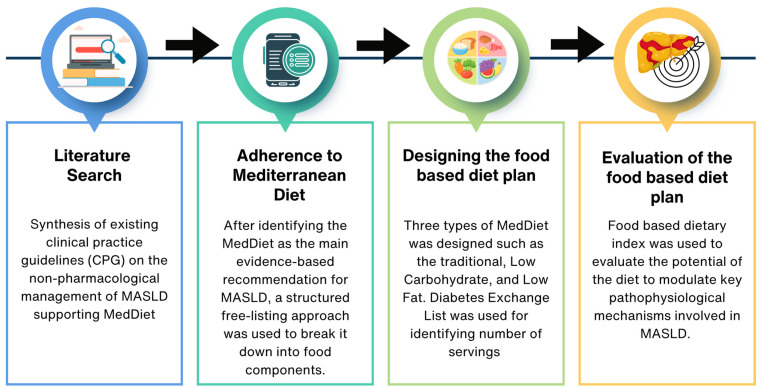
Methodological design for developing a nutritionally optimized food-based diet plan that targets key pathophysiological mechanisms involved in MASLD. Abbreviations: MASLD: Metabolic dysfunction-associated steatotic liver disease; MedDiet: Mediterranean diet.

This study developed a nutritionally optimized, food-based diet plan targeting key pathophysiological mechanisms in MASLD summarized in Figure 1. Clinical guidelines were reviewed, identifying the MedDiet as the evidence-based foundation. Three MedDiet types such as traditional, low-carbohydrate, and low-fat were designed using an exchange list and dietary indices were applied to evaluate their potential to modulate inflammation, oxidative stress, and lipid-related mechanisms.

### 2.1. Synthesis of Existing Clinical Practice Guidelines (CPGs)

A comprehensive literature search was conducted to identify and synthesize current clinical practice guidelines (CPGs) on evidence-based nutrition or non-pharmacological recommendations specific to MASLD. CPGs are essential for enhancing healthcare quality and patient outcomes by enabling clinicians to make timely, evidence-based decisions for patient care [34]. Clinical practice guidelines are essential for dietary recommendations as they synthesize high-quality evidence and expert consensus into clinically validated, safe, and actionable guidance for patient care [35,36]. The literature search was performed to verify that the MedDiet represents the most frequently recommended dietary approach aside from weight loss. Searches were performed using electronic databases, including PubMed/MEDLINE and Google Scholar. Search terms combined controlled vocabulary (“MASLD,” “NAFLD,” “MAFLD,” “non-alcoholic fatty liver disease,” “clinical practice guideline,” “diet,” “nutrition,” “evidence-based recommendation”) with relevant keywords to maximize sensitivity. Clinical practice guidelines were eligible for inclusion if they were official documents and consensus statements issued by recognized scientific societies or health authorities and addressed the management of MASLD, including earlier terminology (NAFLD). We specifically included guidelines that provided explicit recommendations on non-pharmacological management, such as dietary patterns, lifestyle interventions, or weight loss strategies, and that contained actionable guidance such as dietary approaches or specific targets. Documents were excluded if they consisted of primary research studies, narrative reviews, or editorials, focused exclusively on pharmacological or surgical interventions, or lacked specific lifestyle or dietary recommendations. When multiple versions of a guideline were available, the most recent version was included. Only guidelines targeting the adult population were considered. No language restrictions were applied, and the search encompassed publications from database inception through to 2025.

### 2.2. Free Listing and Adherence to Mediterranean Diet

Following the confirmation of the MedDiet pattern as the predominant evidence-based recommendation for MASLD management, a literature-based free-listing approach was employed to operationalize the diet into food components. Free listing was used as a qualitative synthesis method to systematically identify food items consistently described as part of the MedDiet. The analysis focused on peer-reviewed studies and authoritative resources that explicitly defined or applied the MedDiet and reported specific foods [37,38,39,40,41]. Extracted food items were then standardized to consolidate synonyms and variations in terminology; for example, “extra virgin olive oil” and “olive oil” were recorded uniformly as “olive oil.” Standardized foods were subsequently categorized into broad groups, including vegetables, fruits, legumes, cereals/whole grains, nuts and seeds, fish and seafood, dairy, meat/poultry, and oils/fats, enabling consistent comparison across studies and facilitating identification of core MedDiet components.

### 2.3. Nutrient Targets and Dietary Pattern

Three types of MedDiet were designed such as the traditional MedDiet (TMD), low-carbohydrate MedDiet (LCMD) and low-fat MedDiet (LFMD). The three variations in the MedDiet pattern were carefully operationally defined based on their respective macronutrient distributions, allowing for precise comparison of their nutritional profiles. The TMD was formulated to provide approximately 45% of total energy from carbohydrates, 20% from protein, and 35% from total fats, reflecting the classic balance of the Mediterranean dietary pattern. In contrast, the LCMD was specifically designed to reduce carbohydrate intake to around 35% of total energy while increasing the proportion of total fats to 45%, with protein maintained at 20%. Meanwhile, the LFMD aimed to minimize fat intake to roughly 25% of total energy, while increasing carbohydrates to approximately 55%, with protein again contributing 20% of total energy, thus creating a dietary pattern characterized by a lower fat content.

### 2.4. Food Group Classification and Food Group Serving

The Diabetes Exchange List was used to identify the food group classification and food group serving. This tool has six different groups such as starch/bread, meats (very lean, lean, medium-fat, and high-fat), vegetables, fruits, milk (skim, low-fat, and whole) and fats and oil. The six different groups vary in their carbohydrate, protein, fat, and calorie content as shown in Table 1. Each exchange list contains foods that are alike; each food choice on a list contains about the same amount of carbohydrate, protein, fat, and calories as the other choices on that list [42]. In translating portion sizes, it is important to note that one option may represent a larger quantity of food than another option within the same list. Due to the inherent differences among foods, each item is measured or weighed such that the amounts of carbohydrate, protein, fat, and total energy are equivalent across all options.

### 2.5. Evaluation of Dietary Patterns Using Scientific Indexes

To assess the ability of the diet to modulate key pathophysiological mechanisms involved in MASLD, scientific dietary indices including the DII, DAI, DLI, and DLL were calculated for each dietary pattern and comparatively analyzed. A tested Western diet (WD) was used as the positive control and a point of comparison against the TMD.

#### 2.5.1. Dietary Inflammatory Index (DII)

The DII was designed to quantify the pro- or anti-inflammatory properties of dietary intake. The inflammatory potential of each menu was evaluated by calculating the DII. The DII is based on a systematic review of peer-reviewed human, animal and cell culture research studies examining the effects of dietary components including macronutrients, micronutrients, and bioactive compounds on six key inflammatory biomarkers: interleukin-1β (IL-1β), interleukin-4 (IL-4), interleukin-6 (IL-6), interleukin-10 (IL-10), tumor necrosis factor-alpha (TNF-α), and *C*-reactive protein (CRP). DII scores were then calculated as the sum of the standardized and weighted parameters, producing a continuous score reflecting the overall inflammatory potential of the diet. Higher scores (positive values) correspond to more pro-inflammatory diets, while lower scores (negative values) indicate anti-inflammatory diets [28].

Dietary components such as food and nutrient values were derived from the United States Department of Agriculture (USDA) Food Composition Database [43] per 100 g of each food item and subsequently converted to reflect the specific portion sizes. The DII is composed of 45 food parameters such as nutrients, energy, specific food or bioactive compounds. The study excluded alcohol and caffeine as these food parameters are not recommended in the MedDiet context. Additionally, specific polyphenol subclasses, including eugenol, flavan-3-ols, flavones, flavonols, flavanones, anthocyanidins, and isoflavones, were omitted due to the lack of a comprehensive and reliable data source capable of consistently capturing their content across all identified food items. However, due to data availability constraints, the USDA Food Composition Database allows for the calculation of only 36 of these parameters.

- Energy and macronutrients: Energy (kcal), carbohydrate (g), total fat (g), protein (g), fiber (g), saturated fat (g), trans fat (g), MUFA (g), *n*-3 fatty acids (g), *n*-6 fatty acids (g), PUFA (g), and cholesterol (mg).
- Micronutrients: Vitamin B12 (mg), vitamin B6 (mg), β-carotene (µg), folic acid (µg), thiamin (mg), riboflavin (mg), niacin (mg), vitamin A (RE), vitamin C (mg), vitamin D (µg), vitamin E (mg), iron (Fe) (mg), magnesium (Mg) (mg), selenium (Se) (µg), and zinc (Zn) (mg).
- Other food components: Garlic (g), ginger (g), onion (g), saffron (g), turmeric (g), pepper (g), thyme or oregano (g), rosemary (g), and green or black tea (g).

In the present study, only 32 food parameters were included in the analysis. However, for future applications such as menu planning and dietary scoring, all 36 food parameters may be incorporated since the USDA Food Database can calculate these parameters. The framework developed in this study is designed to accommodate the inclusion of all 36 DII parameters available within the database, provided that complete and reliable data are accessible. This ensures that the approach remains scalable, flexible, and adaptable for broader use in subsequent analyses and practical implementations.

#### 2.5.2. Dietary Antioxidant Index (DAI)

The DAI was calculated based on the amount of vitamin A, C, and E, selenium, magnesium, and zinc. The DAI is a valid indicator of dietary antioxidant assessment, and it can be used as a predictor of antioxidant status due to its correlation with serum antioxidant levels [30,44]. We standardized each of the six vitamins and minerals by subtracting the global mean and dividing by the global standard deviation to calculate the DAI. We then calculated the DAI by summing up the standardized intakes of these vitamins and minerals of the individuals with equal weight. The global mean and standard deviation were adopted from the Dietary Inflammatory Index development study [28]. A higher DAI score (positive values) represents a diet rich in antioxidants and is associated with better oxidative balance, while a lower score (negative values) indicates inadequate antioxidant intake and increased disease risk [30,44,45].

#### 2.5.3. Dietary Lipid Index

The Dietary Lipid Index such as DLI and DLL was assessed among the three types of MedDiet pattern. The DLI was calculated by multiplying the amount of specific intake (g) by their melting points (°C), then summing the products and dividing by the total fatty acid intake (g). The DLL was calculated by multiplying the amount of each fatty acid consumed by its melting point, and then summing these values for all fatty acids in the diet [31]. The 22 types of dietary fatty acid were calculated using the USDA Food Composition Database that indicated fatty acid (g) in 100 g of each food and converted it into specific amounts specified in the diet. Dietary fatty acids include saturated fatty acids (SFAs), monounsaturated fatty acids (MUFAs), and polyunsaturated fatty acids (PUFAs), Eicosapentaenoic acid (EPA), Docosahexaenoic acid (DHA) and Docosapentaenoic acid (DPA), and trans fatty acids (TFA). Fatty acid melting point data were obtained from the Lipid Bank database [31,46].

To enable comparison, the original three MedDiet patterns used extra-virgin olive oil (EVOO) as the primary source of dietary fat. For comparative analyses, EVOO was replaced with coconut oil. This substitution allowed us to assess the impact of EVOO on dietary lipid quality.

### 2.6. Western Diet as Point of Comparison

To provide a comparative reference, the study adapted 7-day rotating menus representing a Western diet (WD) or ultra-processed food (UPF) dietary pattern previously used in a randomized controlled trial that reported significant weight gain [47]. The WD was designed to be isocaloric with the TMD and to match its macronutrient composition. This WD or UPF diet serves as a robust benchmark for evaluating the relative health impacts of Mediterranean versus highly processed Western dietary patterns. By including a dietary pattern commonly consumed in modern industrialized settings characterized by high levels of refined carbohydrates, added sugars, and processed fats, the study provides a relevant contrast to the nutrient-rich, minimally processed foods emphasized in the MedDiet. Additionally, this comparison allows for the assessment of whether the selected dietary indices effectively capture differences in diet quality and potential health effects across markedly distinct dietary patterns.

### 2.7. Data Analysis

The DII and DAI were summarized using descriptive statistics (mean ± standard deviation) to characterize the overall profiles and variability of each dietary pattern. The three MedDiet patterns were compared descriptively, while the WD was specifically compared with the TMD. Dietary lipid indices such as the DLI and DLL between the EVOO and coconut oil across the three MedDiets were compared descriptively. All values are presented as mean ± standard deviation (SD) for dietary lipid indices.

## 3. Results

### 3.1. Summary of Clinical Practice Guidelines

A review of international CPG evaluating nutritional management strategies for MASLD was conducted. Guidelines issued by major professional organizations, including the ADA, APWP, EASL–EASD–EASO, AASLD, KASL, NICE and ALEH, were analyzed to identify consensus and divergence in dietary recommendations as shown in Table 2.

Across all reviewed guidelines, dietary modification was consistently identified as a central component of non-pharmacological management for MASLD. Most recommendations emphasized the importance of achieving a sustained caloric deficit, commonly defined as a reduction of 500–750 kcal/day or a total daily energy intake of 1200–1800 kcal/day, to promote weight loss and improve hepatic outcomes. In addition, limiting the intake of saturated fats, refined carbohydrates, fructose, alcohol, and UPFs was uniformly encouraged, alongside increased consumption of whole and minimally processed foods.

Although several dietary approaches including low-carbohydrate diets, ketogenic diets, intermittent fasting, and time-restricted feeding were acknowledged as viable options when adherence and caloric reduction were achieved, the MedDiet pattern emerged as the most consistently recommended approach across guidelines. Multiple organizations explicitly endorsed the MedDiet based on robust evidence demonstrating improvements in hepatic steatosis, insulin sensitivity, systemic inflammation, and cardiometabolic risk factors, effects that may occur independently of weight loss. Collectively, these findings demonstrate substantial agreement among international guidelines and indicate that, while various dietary strategies may be effective, the MedDiet represents the most safe, evidence-supported and widely endorsed dietary pattern for the nutritional management of MASLD.

### 3.2. Adherence to Mediterranean Diet (MedDiet)

The literature-based free-listing method analysis identified a wide range of food items representative of the MedDiet pattern [40,55,56,57,58]. These items were subsequently categorized into major food groups, including whole grains and cereals, legumes and pulses, nuts and oil seeds, fats and oils, vegetables, fruits, herbs and spices, low-fat dairy products, and lean protein sources. The listed foods reflect core components of the MedDiet, with an emphasis on plant-based foods, EVOO as the primary fat source, and regular consumption of fish and other lean meat as the protein source. Table 3 shows the results of free listing of food groups and representative foods included in the MedDiet.

### 3.3. Translation of Macronutrient Distribution to Food Group Serving

The total energy requirement such as 1200 kcal, 1400 kcal, 1600 kcal, 1800 kcal and 2000 kcal was distributed into macronutrient compositions following the three MedDiet patterns. Table 4 shows the exact amounts in grams in each macronutrient following the three MedDiet patterns. This amount was used to determine the number of exchanges allowed in each food group detailed in the food exchange list. Table 5 shows the specific number of servings in each food group in the three MedDiet patterns. A meal pattern was developed for each calorie-specific comprehensive plan, where exchanges were distributed into five meals: three major and two minor meals. An example of one-day sample menu based on 1600 kcal of the three MedDiet patterns is shown in Supplementary Material Table S1.

### 3.4. Evaluation of Dietary Patterns Using Scientific Indexes

#### 3.4.1. Dietary Inflammatory Index Score of the Diet

All MedDiet patterns and WDs were subjected to quantitative analysis, with a total of 32 nutrients and food components computed for each pattern as shown in Table 6. All data are expressed as the mean values derived from 7-day meal plans. All three MedDiet patterns such as the TMD, LFMD, and LCMD demonstrated consistently anti-inflammatory profiles, with average DII scores ranging from −2.69 ± 0.31 to −2.78 ± 0.17. In contrast, the WD exhibited pro-inflammatory characteristics, with a markedly higher average DII score of 5.21 ± 0.31.

**Figure 2:**
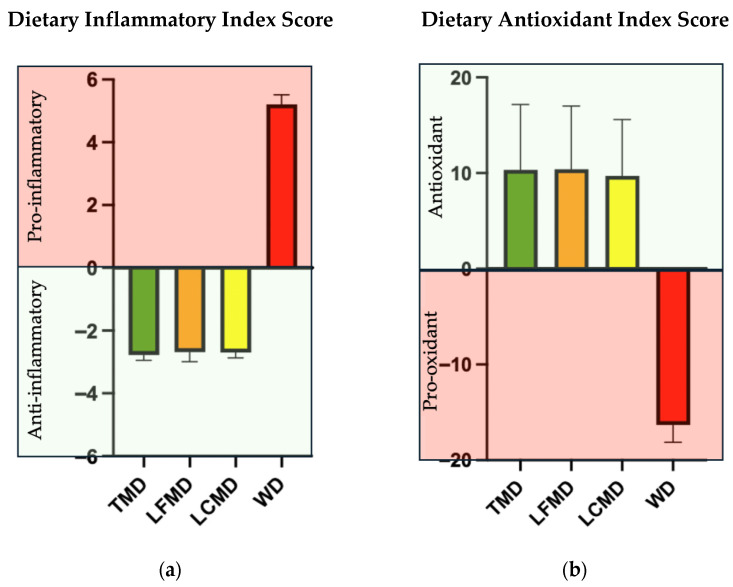
Comparison of the average of (**a**) DII scores and (**b**) DAI scores between the dietary patterns. Abbreviations: TMD: Traditional Mediterranean Diet; LFMD: Low-Fat Mediterranean Diet; LCMD: Low-Carbohydrate Mediterranean Diet: WD: Western Diet.

The DII scores for the 1600 kcal meal plans differed markedly across dietary patterns as shown in Table 7. Daily DII values followed a similar trend, with all Mediterranean patterns maintaining negative scores across all seven days, whereas the WD remained strongly positive. Figure 2a compares the average DII scores across dietary patterns, showing that MedDiets consistently exhibit anti-inflammatory potential, regardless of macronutrient distribution. In contrast, the WD, despite having the same macronutrient composition as the TMD, demonstrates a markedly pro-inflammatory effect.

#### 3.4.2. Dietary Antioxidant Score of the Diet

The DAI scores for the 1600 kcal meal plans across dietary patterns is shown in Table 8. The TMD, LFMD, and LCMD patterns consistently showed positive DAI values, with averages of 10.35 ± 6.81, 10.40 ± 6.61, and 9.72 ± 5.89, respectively. In contrast, the WD consistently yielded negative values, with an average of −16.31 ± 1.81, suggesting a substantially lower antioxidant potential. Daily variations within the TMD, LFMD, and LCMD diets were observed, with peaks on Day 7 (20.03, 18.25, and 17.86, respectively), which may reflect the inclusion of foods with higher antioxidant content on those days, while the WD exhibited a pro-oxidant score of −12.5. Figure 2b compares the average DAI scores across dietary patterns, demonstrating that MedDiets are effective in increasing dietary antioxidants. Despite having similar macronutrient content, the TMD shows markedly higher antioxidant capacity potential than the WD, highlighting that the WD is a pro-oxidant dietary pattern.

#### 3.4.3. Dietary Lipid Index

##### Fatty Acid Composition

The fatty acid composition of the three dietary patterns differed notably depending on the type of fat used, with EVOO and coconut oil showing distinct profiles. Saturated medium-chain fatty acids (MCFAs) such as caprylic (C8:0), capric (C10:0), and lauric acid (C12:0) were substantially higher in diets containing coconut oil compared to EVOO across all patterns, reflecting the naturally high MCFA content of coconut oil. Palmitic (C16:0) and stearic acid (C18:0) were relatively similar between the oils but slightly higher in the LCMD when coconut oil was used. In contrast, MUFAs, particularly oleic acid (C18:1*n*-9), were significantly higher in EVOO-containing diets. PUFAs, including linoleic (C18:2*n*-6) and linolenic acid (C18:3*n*-3), were moderately present in both oils but generally higher in EVOO. Trans fatty acids (16:1T, 18:1T, 18:2T, 22:1T) were negligible in all diets. Overall, the data suggest that the choice of fat source strongly influences the fatty acid profile of a diet: EVOO enriches diets with MUFAs and PUFAs, while coconut oil markedly increases saturated MCFAs. The summary of fatty acid composition of the three dietary patterns can be seen in Table 9.

##### Dietary Lipophilic Index and Dietary Lipophilic Load

The comparison of MedDiet patterns using EVOO versus coconut oil revealed a clear impact of fat source on the DLI. Across all diet types, the DLI values were consistently higher when coconut oil was used, increasing from 22.07 to 22.52 with EVOO to 30.03–32.94 with coconut oil. This increase reflects the higher lipophilic (fat-soluble) content of coconut oil, particularly its rich MCFAs, compared to the MUFA-dominant profile of EVOO. The comparison of MedDiet patterns using EVOO versus coconut oil showed that substituting coconut oil significantly increased the DLL across all diets. For TMD, DLL increased from 1374.59 ± 173.97 with EVOO to 2134.35 ± 334.86 with coconut oil. Similarly, the LFMD and LCMD increased from 1197.00 ± 91.34 to 1584.06 ± 122.31, and from 1514.28 ± 208.79 to 3001.71 ± 980.98, respectively. The largest increase was observed in LCMD, likely due to its higher total fat content and the predominance of MCFAs in coconut oil. Across all MedDiet patterns, replacing EVOO with coconut oil increased both the DLI and DLI as presented in Table 10, highlighting the significant influence of fat source on dietary energy density and lipophilic load.

## 4. Discussion

### 4.1. Macronutrient Composition and MASLD

Macronutrient distribution represents a key component in defining dietary patterns. The role of specific dietary patterns in MASLD remains an area of active investigation. Human intervention studies have examined whether modifications in the quantity and quality of dietary fats and carbohydrates influence the progression of MASLD [61]. In this study, diet plans were developed based on three dietary patterns with differing macronutrient compositions in the context of MedDiet patterns. The variations in macronutrient composition such as low-fat or low-carbohydrate approaches can be achieved primarily through adjustments in food group portions rather than fundamental changes in dietary pattern. Importantly, these modifications do not necessitate a departure from core MedDiet principles, including high intake of vegetables, fruits, whole grains, legumes, and the preferential use of unsaturated fats from plant and marine sources. Emphasizing diet quality, particularly the processing level and the food source of macronutrients, may be more critical than absolute macronutrient distribution in influencing metabolic health outcomes.

Evidence from population studies suggest that excessive intake of certain macronutrients is associated with increased oxidative stress, a pro-inflammatory state, and lipotoxicity, all of which contribute to the development and progression of MASLD [62]. In a Korean cohort, macronutrient composition was identified as a key determinant of MASLD risk. Diets characterized by lower carbohydrate intake and higher consumption of healthy sources of fats and protein were associated with an approximately 10% reduction in the risk of developing MASLD [63]. In a 2-year trial, they explore the effect of low-fat diet (Atkins Diet—30% fat of the total energy), low-carbohydrate diet (American Heart Association) and MedDiet (35% fat of the total energy) on subjects with obesity. They have shown that these three diets provide changes in bilirubin, AST and ALT. The MedDiet and low-carbohydrate diets may be effective alternatives to low-fat diets [64]. The modulation of dietary carbohydrate intake, particularly low carbohydrate consumption, can lower insulin secretion and de novo lipogenesis (DNL) as it reduces blood glucose level [65]. The decrease in DNL can reduce endoplasmic reticulum (ER) stress. A systematic review suggests that a low-carbohydrate diet (approximately 50–140 g daily,) in the context of MASLD, can be shown to reduce liver fat, but direct evidence linking dietary carbohydrate restriction to the mechanisms driving MASH remains limited [61]. Moreover, assessing dietary quality requires consideration of carbohydrate type rather than solely the absolute amount consumed. Recent data indicate that the food form and degree of processing may substantially influence both energy efficiency and the metabolic risk associated with particular carbohydrate sources [66].

Evidence suggests that higher dietary quality is associated with a lower likelihood of developing MASLD and advanced fibrosis [67,68]. Similarly, in the context of type 2 diabetes prevention, the quality of dietary carbohydrate has been shown to be critical, with diets emphasizing plant-based proteins, healthy fats, and high-quality carbohydrates demonstrating the greatest effectiveness.

Across the clinical practice guidelines reviewed, the MedDiet is the most frequently cited and recommended dietary pattern for the prevention and management of MASLD. The MedDiet is characterized by high consumption of unprocessed foods that are high in fiber and healthy fats. Evidence supports its role in improving lipid metabolism, enhancing insulin sensitivity, supporting weight loss, reducing liver stiffness, and potentially reversing histological features of MASLD [17]. Interestingly, in a randomized control trial in MASLD patients [69], they found out that three MedDiet patterns such traditional, low-carbohydrate, and low-fat diets can positively affect MASLD, regardless of macronutrient differences. The study has shown that following either of the three MedDiet patterns significantly decrease BMI and other anthropometric measures such as waist circumference, hip circumference, waist/hip circumference ratio, body fat mass and percentage, abdominal fat mass, and visceral fat. In terms of biochemical parameters, the levels of fasting blood glucose, insulin, insulin resistance, liver enzymes, LDL cholesterol, total cholesterol, total cholesterol/HDL cholesterol ratio, FLI, and FIB-4 scores significantly decreased in the three-diet group. In this study, all types of the MedDiet were compared with a WD, characterized as an UPF dietary pattern. Although the WD provided the same total energy (kcal) as the three MedDiet patterns and a similar macronutrient distribution to the TMD, it showed significantly poorer scores in diet quality indices. These findings suggest that, even when energy intake and macronutrient composition are comparable, dietary quality—particularly the proportion of unprocessed foods—plays a critical role in the dietary management of metabolic diseases, including MASLD. These findings underscore the importance of prioritizing nutrient quality in combination with macronutrient quantity in the prevention and management of metabolic diseases. At the molecular level, the metabolic effects of these variations appear to be driven more by diet quality than by macronutrient quantity alone.

Despite these variations in macronutrients, the menu developed in the study on the three MedDiet patterns has shown that no significant differences were observed in DII and DAI scores. These findings highlight the consistent anti-inflammatory and antioxidant potential of the MedDiet, irrespective of macronutrient distribution. The MedDiet emphasizes unsaturated fatty acids (particularly monounsaturated and omega-3 fatty acids), complex carbohydrates rich in dietary fiber and polyphenols, and high antioxidant content, all of which modulate key pathways involved in MASLD pathogenesis.

### 4.2. Dietary Inflammatory Index and MASLD

The DII analysis provides important insights for the design of therapeutic menu plans for MASLD. Given the pivotal role of chronic inflammation in MASLD pathogenesis [70,71], diets with anti-inflammatory potential are essential [72]. In this study, all MedDiet patterns demonstrated consistently negative DII scores across the seven-day menu cycle, indicating a potential anti-inflammatory profile. Notably, the similarity of DII values among the traditional, low-fat, and low-carbohydrate MedDiet suggests that potential anti-inflammatory benefits can be maintained despite variations in macronutrient composition, offering flexibility in individualized menu planning. In contrast, the WD exhibited markedly higher, positive DII scores, reflecting a strong pro-inflammatory potential that may contribute to hepatic inflammation and disease progression. These findings support the prioritization of MedDiet-based dietary patterns and the avoidance of WD characteristics in the development of menu plans. The DII can be used to assess the inflammatory quality of patients’ habitual diets, guide individualized nutrition therapy, and monitor dietary changes over time. By providing a quantifiable and interpretable measure of potential diet-induced inflammation, the DII may complement existing clinical and biochemical assessments, supporting dietary interventions for MASLD management. Taken together, these findings suggest that the DII is a promising tool for guiding the design of potential anti-inflammatory menu plans with probable relevance for both clinical nutrition practice and broader public health strategies.

### 4.3. Dietary Antioxidant Index and MASLD

The DAI analysis provides additional insight into the design of therapeutic diet plans for MASLD, where oxidative stress plays a central role in disease progression. The consistently positive DAI values observed across the traditional, low-fat, and low-carbohydrate MedDiet patterns indicate a higher antioxidant potential, likely reflecting the regular inclusion of fruits, vegetables, legumes, nuts, and EVOO. Notably, the similarity of DAI values across these patterns suggests that potential antioxidant adequacy can be maintained despite variations in macronutrient composition, offering flexibility in menu planning. In contrast, the WD exhibited markedly negative DAI values, indicating substantially lower antioxidant capacity, which may exacerbate oxidative stress and undermine dietary strategies aimed at liver protection in MASLD. Collectively, these findings support the prioritization of whole-food, MedDiet designs to enhance dietary antioxidant intake in MASLD management.

**Figure 3:**
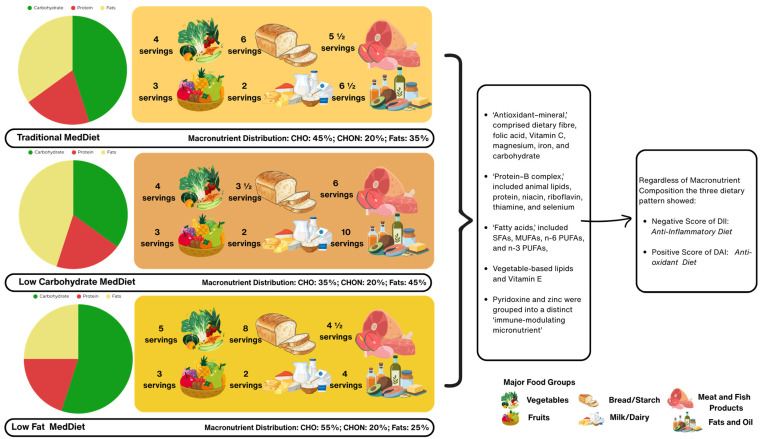
Comparison of the three Mediterranean dietary patterns showing macronutrient distribution and recommended daily servings of major food groups.

Figure 3 shows the comparison of the three Mediterranean dietary patterns with different macronutrient distributions and corresponding recommended daily servings of major food groups. The variations in carbohydrate, protein, and fat intake are reflected in adjusted servings of vegetables, fruits, grains, dairy, meat/fish, and fats/oils, while maintaining the core principles of a MedDiet pattern. The practical importance of comparing traditional, low-fat, and low-carbohydrate MedDiet patterns lies in demonstrating that the potential anti-inflammatory and antioxidant properties of the MedDiet can be preserved despite variations in macronutrient distribution. This finding provides flexibility for individualized dietary prescriptions, allowing clinicians and dietitians to adapt the MedDiet to different metabolic needs, patient preferences, and therapeutic goals while maintaining its beneficial anti-inflammation and antioxidant profile.

### 4.4. Extra-Virgin Olive Oil as the Main Source of Dietary Fat

EVOO, characterized by a high proportion of monounsaturated fatty acids, is consistently associated with favorable lipid and cardiovascular outcomes. In contrast, coconut oil is predominantly composed of SFA, particularly lauric acid, and has been shown to increase LDL cholesterol despite its plant origin. Including coconut oil as a comparator therefore provides a stringent test of the index, ensuring that high SFA content is appropriately reflected regardless of food source, and reinforcing the index’s relevance for evaluating lipid quality rather than origin alone. Dietary MUFAs reduce serum triglyceride levels by promoting fatty acid oxidation through the activation of peroxisome proliferator-activated receptor-α (PPARα) and by suppressing sterol regulatory element-binding protein (SREBP) activity, thereby inhibiting hepatic lipogenesis [73]. In addition, MUFAs activate both PPARα and PPARγ, enhancing lipid oxidation and improving insulin sensitivity, which contributes to a reduction in hepatic steatosis [74]. In the context of MASLD, EVOO has been reported to exert beneficial effects on hepatic steatosis, inflammation, and oxidative stress. EVOO consumption is associated with improvements in liver function parameters, including reductions in serum ALT and AST levels, as well as favorable modulation of circulating lipid profiles and glycemic markers. Furthermore, within the framework of the MedDiet, EVOO intake has been linked to reductions in body weight and body mass index (BMI), which may further contribute to its hepatoprotective effects [15,17]. Beyond their fatty acid profile, olive oil-derived polyphenols play a significant role in lipid metabolism by protecting low-density lipoprotein (LDL) cholesterol from oxidative damage and increasing high-density lipoprotein (HDL) cholesterol levels [75]. Due to its favorable fatty acid composition, primarily the high oleic acid components, EVOO is the only culinary fat associated with potential benefits in weight management [76].

### 4.5. Limitations and Future Directions

This study is based on dietary design and score calculations and does not include direct clinical or biological validation. The approach is informed by established CPG current evidence on MASLD; the findings should be interpreted as providing an evidence-based framework rather than demonstrating clinical efficacy. The study illustrated the potential utility of indices such as the DII and DAI to characterize the inflammatory and antioxidant profiles of dietary patterns. Additionally, dietary lipid indices highlighted the importance of considering the quality of primary dietary fat sources within meal planning. Overall, these findings provide descriptive insights into how different dietary patterns may influence nutritional quality and dietary factors relevant to the prevention and management of MASLD, without implying direct clinical outcomes. Further studies are needed to confirm applicability in real-world or clinical settings.

This study is limited by the use of a qualitative, inclusive approach without predefined thresholds or weighting schemes, which may reduce strict reproducibility in menu construction. However, given emerging evidence showing variability in how the Mediterranean diet is defined and applied particularly in MASLD management outside Mediterranean regions [25], this approach was chosen to preserve flexibility and generalizability. Future work may incorporate quantitative criteria as consensus develops.

A key consideration for the translation of these findings into practice is long-term adherence to the MedDiet. Although our framework integrates nutrient composition with the DII, DAI, DLI and DLL, its effectiveness depends on sustained adherence. Evidence indicates that adherence can be improved through simplified meal preparation, the use of accessible and affordable foods, and adaptation to cultural dietary practices. In addition, structured dietary counseling and behavioral support play an important role in maintaining long-term compliance. The use of digital tools may further support adherence by facilitating personalized meal planning and tracking of dietary indices. Future research should evaluate adherence to such index-based strategies and their impact on long-term MASLD outcomes.

### 4.6. Practical Implications and Digital Translational Potential

This study demonstrates that food-based MedDiet plans can be effectively evaluated using DII, DAI, DLI, and DLL, providing a practical framework to optimize dietary interventions for MASLD. These indices allow healthcare professionals, clinicians and dietitians to quantify anti-inflammatory and antioxidant potential, and lipid quality/quantity. Beyond clinical practice, they can inform digital tools such as meal planning apps or dietary monitoring platforms, offering real-time feedback and adjustments to enhance adherence and therapeutic impact. Importantly, the consistent anti-inflammatory and antioxidant potential across traditional, low-carbohydrate, and low-fat MedDiet patterns highlights flexibility in menu design, enabling culturally appropriate and patient-preferred options. Providing additional research validation and exploration, integrating these indices into routine practice could improve precision, monitoring, and effectiveness of dietary interventions, bridging molecular insights with actionable strategies to prevent or manage MASLD.

**Figure 4:**
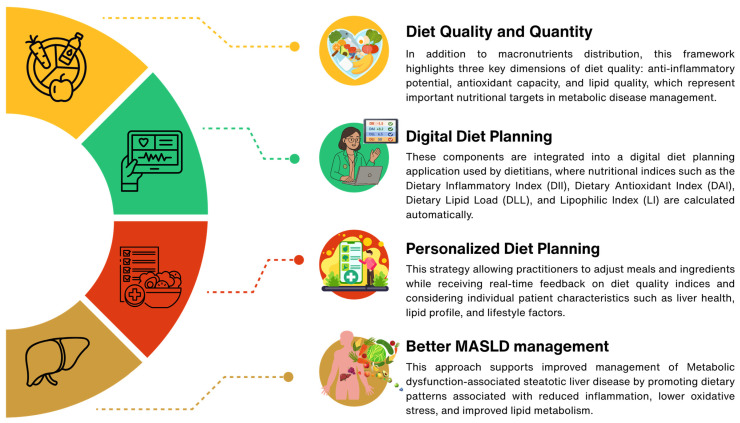
The digital potential for personalized nutrition for MASLD management. Digital diet planning approach integrating diet quality indicators (DII, DAI, DLL, and DLI) and food and nutrient information to support personalized dietary strategies for MASLD.

Food-based dietary strategies are central to the prevention and management of MASLD, as they directly modulate inflammation, oxidative stress, and lipid metabolism. The MedDiet, in its traditional, low-carbohydrate, and low-fat forms, consistently demonstrates anti-inflammatory and antioxidant effects, regardless of macronutrient composition. Nutritional indices such as the DII, DAI, DLI, and DLL provide an evidence-based framework to design and evaluate therapeutic meal plans. This science-based approach could be translated into app-based tools to assist nutrition practitioners in personalizing and monitoring diets as shown in Figure 4. While these findings offer descriptive insights into the potential benefits of MedDiet patterns, human studies are needed to confirm whether tracking these indices can improve health outcomes in patients with MASLD or contribute to disease prevention.

## 5. Conclusions

This study provides a comprehensive evaluation of MedDiet dietary patterns for the nutritional management of MASLD, integrating clinical practice guidelines for non-pharmacological recommendations. The review of international CPGs supports that dietary modification remains a cornerstone of MASLD management, supporting the MedDiet as the most evidence-based dietary pattern due to its beneficial effects on hepatic steatosis, insulin sensitivity, inflammation, and cardiometabolic risk.

The development of calorie-specific meal plans demonstrated that variations in macronutrient distribution such as traditional, low-fat, and low-carbohydrate MedDiet patterns can be successfully implemented through adjustments in food group servings while maintaining the fundamental principles of the MedDiet pattern. Importantly, the evaluation using dietary indices revealed that all MedDiet types maintained consistently favorable profiles, characterized by anti-inflammatory potential (negative DII scores) and antioxidant capacity potential (positive DAI scores). In contrast, the WD exhibited markedly potential pro-inflammatory and pro-oxidative characteristics despite similar calorie and macronutrient composition with the TMD highlighting the critical role of dietary quality and food processing level beyond macronutrient quantity alone. Furthermore, the analysis of lipid-related indices underscored the importance of fat quality within MedDiet patterns. Diets based on EVOO demonstrated more favorable types of fatty acid or lipid profiles, mainly MUFAs. These findings reinforce the central role of EVOO as the principal dietary fat within MedDiet frameworks for MASLD management.

Collectively, the results emphasize that diet quality, particularly the emphasis on minimally processed, plant-rich foods and healthy fats, is a key determinant of the therapeutic potential of dietary interventions for MASLD. The integration of dietary indices such as the DII, DAI, DLI, and DLL provides a practical and quantitative framework for evaluating and designing evidence-based therapeutic meal plans. Indices such as the DII and DAI provide a framework for meal planning by helping determine whether a given meal plan aligns with potential anti-inflammatory and antioxidant principles. This will help to target key pathological mechanisms of MASLD such as inflammation and oxidative stress. Also, dietary lipid indices such as the DLI and DLL can be added as guidance in choosing the main dietary fat source in the diet. Exploring the integration of these indices into digital nutrition platforms to enhance accessibility could be a valuable direction for future research. Additional research is warranted to strengthen the evidence base and assess the clinical utility of these index-guided dietary frameworks.

## Figures and Tables

**Table 1:** Food exchange list.

Food Group	Carbohydrate (g)	Protein (g)	Fat (g)	Calories
I. Starch/Bread	15	3	trace	80
II. Meat				
Very Lean	–	7	0–1	35
Lean	–	7	3	55
Medium-Fat	–	7	5	75
High-Fat	–	7	8	100
III. Vegetable	5	2	–	25
IV. Fruit	15	–	–	60
V. Milk				
Skim	12	8	0–3	90
Low-fat	12	8	5	120
Whole	12	8	8	150
VI. Fat	–	–	5	45

**Table 2:** Summary of clinical practice guidelines related to non-pharmacological treatment for MASLD.

Clinical Practice Guidelines	Weight Loss Recommendation	Food- & Nutrient-Related Recommendations	Dietary Pattern Recommendations
ADA (American Diabetes Association) (2025) [48]	≥5% weight loss decreases steatosis; >5% usually required to reverse steatohepatitis	Limit saturated fat, sucrose, high-fructose corn syrup, simple sugars, and ultra-processed foods; increase fiber and unsaturated fats	Mediterranean diet preferred (rich in fruits, vegetables, whole grains, heart-healthy fats)
APWP (Asian Pacific Working Party) (2018) [49]	Target ~10% weight loss; some improvement seen with 3–5%; gradual loss ≤ 1 kg/week with 500–1000 kcal deficit	Avoid energy-dense foods high in saturated fat, cholesterol, and sugar-sweetened beverages; increase fruits, fiber, green vegetables, omega-3 PUFA	Several options proposed: low-carbohydrate, low-fat, and Mediterranean diets
EASL (Europheean Association for the Study of the Liver) (2024) [50]	>5% weight loss reduces liver fat; 7–10% improves inflammation; >10% improves fibrosis	Limit ultra-processed foods, sugars, saturated fats, and sugar-sweetened beverages	Improve diet quality similar to Mediterranean diet; increase unprocessed/minimally processed foods
AASLD (American Association for the Study of Liver Diseases) (2023) [51]	3–5% weight loss improves steatosis; >10% usually required to improve NASH and fibrosis	Avoid excess calories, saturated fats, refined carbs, and sugar-sweetened beverages; limit fructose; coffee may be beneficial	Multiple approaches effective (low-carb, low-fat, intermittent fasting, Mediterranean); Mediterranean commonly recommended
KASL (Korean Association for the Study of the Liver) (2021) [52]	>5–7% weight loss reduces intrahepatic fat and inflammation; 3–5% beneficial in non-obese NAFLD; gradual loss ≤ 1 kg/week recommended	High carbohydrate and fructose intake associated with fatty liver and elevated liver enzymes	Calorie control (≈1500–1800 kcal men, 1200–1500 kcal women); low-carb and low-fat diets both effective; Mediterranean diet improves insulin resistance and liver fat
NICE (National Institute for Health and Care Excellence) (2016) [53]	Focus on weight management in overweight/obese patients	Not specified in detail; aligned with obesity management guidance	Low-energy diets (800–1200 kcal/day) or very-low-energy diets (<800 kcal/day) effective with ongoing support
ALEH (Latin American Association for the Study of the Liver) (2025) [54]	7–10% weight loss recommended (10% in at-risk MASH); 3–5% beneficial for lean MASLD	Reduce overall calories; avoid foods high in saturated and trans fats	Mediterranean diet emphasized due to high monounsaturated fats and omega-3s; improves insulin sensitivity and reduces liver fat

Abbreviations: ADA: American Diabetes Association; APWP: Asia Pacific Working Party on Non-Alcoholic Fatty Liver Disease; EASL–EASD–EASO: European Association for the Study of the Liver, European Association for the Study of Diabetes, European Association for the Study of Obesity; AASLD: American Association for the Study of Liver Diseases; KASL: Korean Association for the Study of the Liver; NICE: National Institute for Health and Care Excellence; ALEH: Latin American Association for the Study of the Liver.

**Table 3:** Food groups and representative foods included in the MedDiet.

Recommendation as per Mediterranean Dietary Guidelines [59]	Food Groups	Listing of Recommended Food Items Found in MedDiet
Include whole grains and cereals	Whole grains and cereals	Barley, Bread, Buckwheat, Bulgur, Couscous, Durum, Farro, Freekeh, Millet, Oats, Orzo, Pasta, Pita bread, Polenta, Quinoa, Rice, Whole wheat
Include whole legumes and pulses	Whole legumes and pulses	Black beans, Black-eyed beans, Chickpeas, Kidney beans, Lentils, Split peas
Include nuts and oilseeds in the diet	Nuts and oil seeds	Almonds, Hazelnuts, Peanuts, Pine nuts, Pistachios, Pumpkin seeds, Sesame seeds, Sunflower seeds, Walnuts, Tahini, Flax seeds, Fennel seeds
Add good fats and oils in diet	Fats and oils	Extra-virgin olive oil (EVOO)
Add a variety of vegetables in your diet	Vegetable	Artichokes, Corn, Cucumbers, Eggplant, Fennel, Garlic, Green beans, Leafy greens, Leeks, Mushrooms, Onions, Peas, Peppers, Potatoes, Root vegetables, Spinach, Squash, Tomatoes, Zucchini
Add a variety of fruits in your diet	Fruits	Apple, Apricots, Avocados, Cherries, Clementines, Dates, Figs, Grapefruit, Grapes, Honeydew, Kiwi, Lemons, Limes, Melons, Nectarines, Olives, Oranges, Peaches, Pears, Plums, Pomegranates, Strawberries, Tangerines, Tomatoes Dried: Apricots, Blueberries, Cherries, Cranberries, Figs, Prunes, Raisins
Include a variety of spices and condiments in your diet	Herbs and spices	Basil, Bay leaf, Black pepper, Cloves, Coriander, Cumin, Dill, Fennel, Garlic, Lavender, Marjoram, Mint, Oregano, Paprika, Parsley, Rosemary, Saffron, Sage, Savory, Sumac, Tarragon, Thyme, Turmeric
Add low-fat dairy products	Low-fat dairy products	Cheese (in moderation), Curd, Milk, Milk products
Add lean meats, eggs and fish and avoid red meat in your diet	Lean meats	Chicken, Chicken eggs, Fish (Mackerel, Salmon, Sardines, Tuna)

**Table 4:** Macronutrient distribution across the three MedDiet patterns at different energy levels.

Total Energy	Traditional MedDiet			Low-Carbohydrate MedDiet			Low-Fat MedDiet		
	CHO (g)	CHON (g)	Fats (g)	CHO (g)	CHON (g)	Fats (g)	CHO (g)	CHON (g)	Fats (g)
1200 kcal	135	60	50	105	60	60	165	60	35
1400 kcal	160	70	55	125	70	70	200	70	40
1600 kcal	180	80	60	140	80	80	220	80	45
1800 kcal	200	90	70	160	90	90	250	90	50
2000 kcal	225	100	80	175	100	100	275	100	60

Abbreviations: kcal: kilocalorie; g: grams; CHO: carbohydrates; CHON: protein; kcal: kilocalories.

**Table 5:** Serving distribution for calorie-specific meal plans across the three MedDiet patterns at different energy levels.

Types of Diet	Total Energy	Number of Serving/s in Each Major Food Group						
		Vegetable	Fruits	Milk/Dairy	Starch/Bread	Low-Fat Meat	Medium-Fat Meat	Fats/Oil
TMD	1200 kcal	3	3	1	4	5	0	5
	1400 kcal	3	3	1	6	4 ½	1	6
	1600 kcal	4	3	2	6	4 ½	1	6 ½
	1800 kcal	4	3	2	7 ½	4	2	6 ½
	2000 kcal	4	3	2	9	5	2	9
LCMD	1200 kcal	4	3	1	2	6	0	7 ½
	1400 kcal	4	3	1	3	5	1 ½	8 ½
	1600 kcal	4	3	2	3 ½	5	1	10
	1800 kcal	4	3	2	5	5	2	11
	2000 kcal	4	3	2	6	6	2	12 ½
LFMD	1200 kcal	4	3	1	6	4	0	3 ½
	1400 kcal	4	3	2	7	2	1 ½	3 ½
	1600 kcal	5	3	2	8	3 ½	1	4
	1800 kcal	5	4	2	9	3	2	4
	2000 kcal	5	5	2	10	4	2	4 ½

Approximation of 1 serving: Vegetables: ½ cup cooked or 1 cup raw; Fruit: 1 small fruit, ½ cup canned/fresh fruit, or ¼ cup dried; Milk (fat-free/low-fat): 1 cup (8 oz); Starch: 1 slice bread, ½ cup cooked cereal, or ⅓ cup rice or pasta; Meat (Lean); 1 oz; Meat (medium-fat): 1 oz; Fat: 1 tsp oil, mayo, or 1 tbsp dressing. These are just estimations of common food items. Refer to the exchange list for other food items [42,60]. Abbreviations: TMD: Traditional Mediterranean Diet; LFMD: Low-Fat Mediterranean Diet; LCMD: Low-Carbohydrate Mediterranean Diet; kcal: Kilocalories.

**Table 6:** Food and nutrient components of the three types of Mediterranean and Western diets presented as mean ± SD based on the 1600 kcal energy computation.

Food or Nutrient	TMD (Mean ± SD)	LCMD (Mean ± SD)	LFMD (Mean ± SD)	WD (Mean ± SD)
Energy (kcal)	1615.0 ± 10	1600.0 ± 5	1648.0 ± 12	1625 ±10
Carbohydrate (g)	179.0 ± 5	142.0 ± 3	217.0 ± 4	243.53 ± 5
Total Fat (g)	61.0 ± 3	80.0 ± 5	46.0 ± 5	73.81 ± 0
Protein (g)	80.6 ± 2	77.0 ± 8	81.0 ± 4	72.71 ± 11.40
Fiber (g)	36.1 ± 11.4	34.8 ± 10.6	40.6 ± 12.6	19.52 ± 1.69
Saturated Fat (g)	13.5 ± 2.8	14.6 ± 3.1	11.7 ± 1.6	24.50 ± 5.64
Trans Fat (g)	0.05 ± 0.06	0.06 ± 0.07	0.05 ± 0.07	1.51 ± 2.44
MUFA (g)	28.1 ± 3.3	32.1 ± 4.0	24.6 ± 1.7	4.39 ± 2.97
*n*-3 Fatty Acids (g)	4.8 ± 4.6	6.2 ± 4.1	3.4 ± 5.1	0.12 ± 0.10
*n*-6 Fatty Acids (g)	12.1 ± 2.7	14.1 ± 3.5	11.0 ± 2.3	0.52 ± 0.65
PUFA (g)	17.8 ± 6.2	21.6 ± 5.5	14.8 ± 6.8	3.46 ± 3.17
Cholesterol (mg)	283.7 ± 144.9	297.9 ± 144.7	248.4 ± 129.6	159.18 ± 51.65
Vitamin B12 (mg)	7.1 ± 4.9	7.8 ± 5.9	6.0 ± 4.1	0.39 ± 0.34
Vitamin B6 (mg)	2.2 ± 0.5	2.3 ± 0.5	2.2 ± 0.4	0.12 ± 0.11
β-Carotene (µg)	3778.4 ± 2804.5	3783.7 ± 2803.4	3637.6 ± 3017.7	27.22 ± 38.49
Folic Acid (µg)	30.0 ± 18.4	22.9 ± 19.0	38.7 ± 22.9	21.64 ± 21.80
Thiamin (mg)	1.6 ± 0.3	1.4 ± 0.4	1.7 ± 0.2	0.19 ± 0.20
Riboflavin (mg)	2.4 ± 0.9	2.2 ± 0.5	2.4 ± 0.9	0.21 ± 0.11
Niacin (mg)	24.9 ± 5.6	24.7 ± 5.9	24.2 ± 5.8	1.70 ± 1.28
Vitamin A (RE)	1297.0 ± 470.4	1331.0 ± 518.7	1396.5 ± 688.0	734.88 ± 241.53
Vitamin C (mg)	128.9 ± 39.5	130.2 ± 39.5	128.5 ± 39.7	48.71 ± 96.16
Vitamin D (µg)	5.91 ± 0.74	5.97 ± 0.75	5.44 ± 1.35	1.65 ± 1.26
Vitamin E (mg)	14.5 ± 8.1	15.4 ± 7.9	14.3 ± 8.4	0.18 ± 0.17
Iron (mg)	14.6 ± 2.41	12.89 ± 2.05	16.23 ± 1.93	7.77 ± 1.15
Magnesium (mg)	475.6 ± 82.0	436.2 ± 74.9	525.0 ± 111.0	27.73 ± 13.94
Selenium (µg)	151.6 ± 53.0	142.7 ± 48.8	149.9 ± 57.9	8.71 ± 9.56
Zinc (mg)	11.4 ± 1.8	10.7 ± 2.0	12.1 ± 2.0	1.08 ± 0.79
Garlic (g)	4.3 ± 0	5.0 ± 0	5.7 ± 1.9	0
Onion (g)	21.4 ± 8.4	24.3 ± 7.9	21.4 ± 8.4	0
Turmeric (g)	5.0 ± 0	5.0 ± 0	5.0 ± 0	0
Thyme/Oregano (g)	5.0 ± 0	5.0 ± 0	5.0 ± 0	0
Green/Black Tea (g)	5.0 ± 0	5.0 ± 0	5.0 ± 0	0

SD: Standard deviation; kcal: Kilocalories; g: grams; mg: milligrams; µg: micrograms; MUFA: Monounsaturated fatty acids; PUFA: Polyunsaturated fatty acids; *n*-3: Omega-3 fatty acids; *n*-6: Omega-6 fatty acids; TMD: Traditional Mediterranean Diet; LFMD: Low-Fat Mediterranean Diet; LCMD: Low-Carbohydrate Mediterranean Diet: WD: Western diet. All values are expressed as mean ± standard deviation.

**Table 7:** Dietary Inflammatory Index (DII) score based on the 1600 kcal computation.

Menu	Types of Dietary Pattern			
	TMD	LFMD	LCMD	WD
Day 1	−2.52	−2.00	−2.50	5.34
Day 2	−2.13	−2.14	−2.28	5.86
Day 3	−2.18	−2.21	−2.29	5.60
Day 4	−2.39	−2.22	−2.68	6.09
Day 5	−2.81	−2.47	−2.55	5.57
Day 6	−2.44	−2.60	−2.56	5.00
Day 7	−2.53	−2.44	−2.29	5.00
Average ± SD	−**2.43 ± 0.23**	−**2.30 ± 0.21**	−**2.45 ± 0.16**	**5.49 ± 0.41**

TMD: Traditional Mediterranean Diet; LFMD: Low-Fat Mediterranean Diet; LCMD: Low-Carbohydrate Mediterranean Diet: WD: Western Diet. (+) values defined as pro-inflammatory; (−) value defined as anti-inflammatory.

**Table 8:** Dietary Antioxidant Index (DAI) score based on the 1600 kcal computation.

Menu	Types of Dietary Pattern			
	TMD	LFMD	LCMD	WD
Day 1	4.07	4.88	5.38	−16.96
Day 2	13.68	14.25	12.63	−16.61
Day 3	4.56	4.14	5.67	−17.12
Day 4	17.9	18.46	16.49	−17.99
Day 5	4.15	3.28	3.05	−15.88
Day 6	8.04	9.52	6.94	−17.15
Day 7	20.03	18.25	17.86	−12.47
Average ± SD	**10.35 ± 6.81**	**10.40 ± 6.61**	**9.72 ± 5.89**	−**16.31 ± 1.81**

TMD: Traditional Mediterranean Diet; LFMD: Low-Fat Mediterranean Diet; LCMD: Low-Carbohydrate Mediterranean Diet: WD: Western Diet. (+) values defined as pro-oxidant; (−) values defined as antioxidant.

**Table 9:** Fatty acid composition of dietary patterns using EVOO and coconut oil presented as mean ± SD based on the 1600 kcal energy computation.

Types of Fatty Acid	TMD		LFMD		LCMD	
	EVOO *	Coconut Oil **	EVOO *	Coconut Oil **	EVOO *	Coconut Oil **
6:0, Caproic acid	0.116 ± 0.06	0.257 ± 0.08	0.105 ± 0.048	0.198 ± 0.046	0.115 ± 0.06	0.350 ± 0.14
8:0, Caprylic acid	0.093 ± 0.04	2.233 ± 0.51	0.082 ± 0.031	1.491 ± 0.133	0.105 ± 0.05	3.649 ± 1.64
10:0, Capric acid	0.246 ± 0.13	1.948 ± 0.49	0.203 ± 0.065	1.320 ± 0.109	0.238 ± 0.13	3.063 ± 1.38
12:0, Lauric acid	0.209 ± 0.10	13.406 ± 3.10	0.189 ± 0.076	8.848 ± 0.734	0.209 ± 0.10	22.123 ± 10.07
14:0, Myristic acid	0.989 ± 0.33	6.260 ± 1.36	0.832 ± 0.250	4.291 ± 0.385	1.031 ± 0.31	9.783 ± 4.13
16:0, Palmitic acid	9.678 ± 1.38	9.963 ± 1.38	8.403 ± 0.792	8.048 ± 0.759	10.572 ± 1.62	12.150 ± 2.43
18:0, Stearic acid	3.282 ± 0.72	3.383 ± 0.62	2.823 ± 0.305	2.726 ± 0.285	3.596 ± 0.92	4.129 ± 0.89
16:1*n*-7, Palmitoleic acid	1.008 ± 0.18	0.792 ± 0.17	0.793 ± 0.113	0.639 ± 0.105	1.195 ± 0.25	0.859 ± 0.17
18:1*n*-9, Oleic acid	27.796 ± 2.52	13.957 ± 2.34	24.657 ± 1.312	11.483 ± 1.198	31.455 ± 3.49	16.610 ± 3.31
20:1*n*-9, Eicosanoic acid	0.480 ± 0.31	0.433 ± 0.28	0.392 ± 0.242	0.338 ± 0.216	0.520 ± 0.32	0.472 ± 0.29
22:1*n*-11, Cetoleic acid	0.101 ± 0.20	0.100 ± 0.19	0.090 ± 0.157	0.090 ± 0.145	0.090 ± 0.19	0.090 ± 0.18
18:2*n*-6, Linoleic acid	12.262 ± 2.34	10.881 ± 2.33	10.337 ± 1.549	9.153 ± 1.252	14.467 ± 3.37	12.566 ± 3.63
18:3*n*-3, Linolenic acid	4.515 ± 4.67	3.221 ± 4.74	3.095 ± 5.209	2.952 ± 4.821	6.038 ± 4.26	3.584 ± 4.64
18:4*n*-6, Octadecatetraenoic acid	0.029 ± 0.04	0.029 ± 0.04	0.021 ± 0.028	0.021 ± 0.026	0.032 ± 0.04	0.032 ± 0.04
20:4*n*-6, Arachidonic acid	0.243 ± 0.15	0.243 ± 0.14	0.193 ± 0.096	0.193 ± 0.089	0.262 ± 0.16	0.262 ± 0.15
20:5*n*-3, Eicosapentaenoic acid	0.231 ± 0.20	0.231 ± 0.19	0.179 ± 0.158	0.179 ± 0.146	0.265 ± 0.24	0.265 ± 0.22
22:5*n*-3, Docosapentaenoic acid	0.203 ± 0.34	0.203 ± 0.31	0.159 ± 0.272	0.159 ± 0.252	0.239 ± 0.41	0.239 ± 0.38
22:6*n*-3, Docosahexaenoic acid	0.723 ± 0.49	0.723 ± 0.46	0.564 ± 0.390	0.564 ± 0.362	0.809 ± 0.59	0.809 ± 0.55
16:1T	0.004 ± 0.01	0.003 ± 0.01	0.002 ± 0.004	0.002 ± 0.004	0.004 ± 0.01	0.004 ± 0.01
18:1T	0.028 ± 0.07	0.034 ± 0.07	0.014 ± 0.035	0.019 ± 0.032	0.029 ± 0.07	0.039 ± 0.07
18:2T	0.028 ± 0.07	0.029 ± 0.07	0.013 ± 0.035	0.015 ± 0.033	0.029 ± 0.07	0.031 ± 0.07
22:1T	0.016 ± 0.04	0.016 ± 0.04	0.016 ± 0.042	0.016 ± 0.039	0.017 ± 0.04	0.016 ± 0.04

The calculation of all fatty acid types was based on the overall diet, * in which extra-virgin olive oil (EVOO) was the primary source of dietary fat, ** in which coconut oil was the primary source of dietary fat. All values are expressed as mean ± standard deviation. Abbreviations: TMD: Traditional Mediterranean Diet; LFMD: Low-Fat Mediterranean Diet; LCMD: Low-Carbohydrate Mediterranean Diet 16:1T (palmitoleic acid/trans-9-Hexadecenoic acid); 18:1T (Octadecenoic acid/Octadecylenic acid); 18:2T (trans-8, trans-10-Octadecadienoic acid); 22:1T (Docosenoicacid/Brassidic acid).

**Table 10:** Dietary Lipid Index in three dietary patterns using EVOO versus coconut oil.

Types of MD Diet	Dietary Lipophilic Index (DLI)		Dietary Lipophilic Load (DLL)	
	EVOO ^+^	Coconut Oil ^++^	EVOO ^+^	Coconut Oil ^++^
TMD	22.072 ± 1.926	31.229 ± 2.759	1374.59 ± 173.97	2134.35 ± 334.86
LFMD	22.516 ± 2.071	30.033 ± 2.354	1197.00 ± 91.34	1584.06 ± 122.31
LCMD	21.234 ± 1.637	32.940 ± 2.871	1514.28 ± 208.79	3001.71 ± 980.98

^+^ The calculation of all fatty acid types was based on the overall diet, in which extra-virgin olive oil (EVOO) was the primary source of dietary fat. ^++^ The calculation of all fatty acid types was based on the overall diet, in which coconut oil was the primary source of dietary fat. All values are expressed as mean ± standard deviation.

## Data Availability

The original contributions presented in this study are included in the article/Supplementary Materials. Further inquiries can be directed to the corresponding authors.

## Supplementary Materials

The following supporting information can be downloaded at: https://www.mdpi.com/article/10.3390/nu18081281/s1, Table S1: One-day sample menu (1600 kcal) by gram weight: Three types of Mediterranean Diet.

## Abbreviations

The following abbreviations are used in this manuscript:

ASLD	American Association for the Study of Liver Diseases
ADA	American Diabetes Association
ALEH	Latin American Association for the Study of the Liver
APWP	Asia Pacific Working Party on Non-Alcoholic Fatty Liver Disease
BMI	Body Mass Index
CPGs	Clinical Practice Guidelines
CRP	C-Reactive Protein
DAI	Dietary Antioxidant Index
DASH	Dietary Approach to Stop Hypertension
DHA	Docosahexaenoic Acid
DII	Dietary Inflammatory Index
DLL	Dietary Lipophilic Load
DLI	Dietary Lipophilic Index
DNL	De Novo Lipogenesis
DPA	Docosapentaenoic Acid
EASL	European Association for the Study of the Liver
EASD	European Association for the Study of Diabetes
EASO	European Association for the Study of Obesity
EPA	Eicosapentaenoic Acid
ER	Endoplasmic Reticulum
EVOO	Extra-Virgin Olive Oil
IF	Intermittent Fasting
IL-1β	Interleukin-1 beta
IL-4	Interleukin-4
IL-6	Interleukin-6
IL-10	Interleukin-10
KASL	Korean Association for the Study of the Liver
KD	Ketogenic Diet
LCD	Low Carbohydrate Diet
LFD	Low Fat Diet
MASLD	Metabolic Dysfunction-Associated Steatotic Liver Disease
MCFAs	Medium-Chain Fatty Acids
MedDiet	Mediterranean Diet
MUFA	Monounsaturated Fatty Acids
NAFLD	Non-Alcoholic Fatty Liver Disease
NICE	National Institute for Health and Care Excellence
PUFA	Polyunsaturated Fatty Acids
ROS	Reactive Oxygen Species
SFA	Saturated Fatty Acids
T2DM	Type 2 Diabetes Mellitus
TFA	Trans Fatty Acids
TNF-α	Tumor Necrosis Factor Alpha
UPF	Ultra-Processed Food
USDA	United States Department of Agriculture
VD	Vegetarian Diet
WC	Waist Circumference
WD	Western Diet
