# Supplementary material for: A Descriptive Analysis of Mediterranean Diet Meal Plans Using the Dietary Inflammatory Index, Dietary Antioxidant Index, and Dietary Lipid Indices: Implications for Dietary Intervention for Metabolic Dysfunction-Associated Steatotic Liver Disease (MASLD) Research
Source: Nutrients. 2026 Apr 17;18(8):1281. doi: 10.3390/nu18081281 (PMC13118456; doi:10.3390/nu18081281)
Supplement: Supplementary file 1 [file nutrients-18-01281-s001.zip › nutrients-4236939-supplementary.pdf]

Dietary Strategies for the Prevention and Management of Metabolic Dysfunction–Associated Steatotic Liver Disease (MASLD): A Scientific Approach Integrating Nutrient Composition, Dietary Antioxidant Index, Dietary Lipid Index, and Dietary Inflammatory Index

Table S1. One-day sample menu (1600 kcal) by gram weight: Three types of Mediterranean Diet.

	TMD	LFMD	LCMD
**Breakfast**	**Weight (grams)**	**Weight (grams)**	**Weight (grams)**
Rolled oats	40	80	20
Blueberries	85	85	85
Boiled egg white	100	50	100
Flaxseed	5		10
Low-fat milk	240	240	240
Cinnamon	5	5	5
**Snack**			
Apple	80	80	80
Almonds	2		12
**Lunch**			
Whole wheat fusilli (cooked)	80	120	40
Tofu	110	110	110
Eggplant	180	180	180
Tomato puree	20	20	20
Onion	30	30	30
EVOO	10	10	10
Garlic powder	5	5	5
Basil	5	5	5
**Snack**			
Whole wheat bread	30	45	30
Peanut butter	5		5
Plain, yogurt low fat	240	240	240
**Dinner**			
Tuna, steamed	100	80	120
Barley (cooked)	40	40	20
Chickpeas	30	30	15
Pumpkin	90	90	90
Carrots	90	90	90
Onion	30	30	30
Celery	30	30	30
EVOO	10	10	10
Thyme	5	5	5
Chives	5	5	5
Turmeric	5	5	5

Orange	80	80	80
Green Tea/Black Tea	5	5	5

Abbreviations: TMD: Traditional Mediterranean Diet; LFMD: Low fat Mediterranean Diet; LCMD: Low Carbohydrate Mediter-ranean Diet;
